# Dual-Task Interference in a Simulated Driving Environment: Serial or Parallel Processing?

**DOI:** 10.3389/fpsyg.2020.579876

**Published:** 2021-01-12

**Authors:** Mojtaba Abbas-Zadeh, Gholam-Ali Hossein-Zadeh, Maryam Vaziri-Pashkam

**Affiliations:** ^1^School of Cognitive Sciences, Institute for Research in Fundamental Sciences (IPM), Tehran, Iran; ^2^School of Electrical and Computer Engineering, College of Engineering, University of Tehran, Tehran, Iran; ^3^Laboratory of Brain and Cognition, National Institute of Mental Health, Bethesda, MD, United States

**Keywords:** dual-task interference, driving, drift diffusion model, task order predictability, dual-task theories

## Abstract

When humans are required to perform two or more tasks concurrently, their performance declines as the tasks get closer together in time. Here, we investigated the mechanisms of this cognitive performance decline using a dual-task paradigm in a simulated driving environment, and using drift-diffusion modeling, examined if the two tasks are processed in a serial or a parallel manner. Participants performed a lane change task, along with an image discrimination task. We systematically varied the time difference between the onset of the two tasks (Stimulus Onset Asynchrony, SOA) and measured its effect on the amount of dual-task interference. Results showed that the reaction times (RTs) of the two tasks in the dual-task condition were higher than those in the single-task condition. SOA influenced the RTs of both tasks when they were presented second and the RTs of the image discrimination task when it was presented first. Results of drift-diffusion modeling indicated that dual-task performance affects both the rate of evidence accumulation and the delays outside the evidence accumulation period. These results suggest that a hybrid model containing features of both parallel and serial processing best accounts for the results. Next, manipulating the predictability of the order of the two tasks, we showed that in unpredictable conditions, the order of the response to the two tasks changes, causing attenuation in the effect of SOA. Together, our findings suggest higher-level executive functions are involved in managing the resources and controlling the processing of the tasks during dual-task performance in naturalistic settings.

## Introduction

Humans have limited cognitive capacity. They can only attend to a few items in the scene (Pylyshyn and Storm, 1988; Huang et al., 2007), maintain and manipulate a few items in working memory (Kane and Engle, 2000; Engle, 2002), have limits in the amount of information they can store in short and long term memory (Anderson et al., 1996), and their performance is hindered when they are asked to handle multiple demands in close temporal proximity (Pashler, 1994a). One of the manifestations of this limited capacity is dual-task interference. When performing two tasks concurrently, reaction times increase and accuracies decrease as the two tasks get close together in time (Pashler and Johnston, 1989). During driving, this phenomenon manifests itself in performance declines when drivers attempt to drive and perform a secondary task simultaneously (Horrey and Wickens, 2004; Blanco et al., 2006; Strayer et al., 2017). Despite the importance of dual-task interference in everyday tasks such as driving and its potentially fatal consequences (Bakhit et al., 2018), most studies of dual-task interference have used artificial paradigms to investigate the underlying mechanisms of dual-task interference (Sigman and Dehaene, 2005, 2008; Miller et al., 2009). In this study, taking the artificial designs one step closer to the natural task of driving, we aim to examine the underlying mechanisms of dual-task interference in a simulated driving environment.

To systematically investigate dual-task interference in artificial tasks (Pashler and Johnston, 1989; Pashler, 1994a), the time interval between the onsets of the first and the second stimulus (henceforth referred to as the Stimulus Onset Asynchrony or SOA) has been varied. It has been shown that when the SOA decreases, the RTs increase and the accuracies decrease. This performance decline as a function of SOA has been used as a measure of dual-task interference. A couple of studies using a simulated driving environment have shown similar effects of SOA on dual-task interference (Levy et al., 2006; Hibberd et al., 2013). These studies provide evidence for dual-task interference in driving, but they do not shed light on its underlying mechanisms.

Several theories have been proposed to explain the dual-task interference; the two most influential of them are the “bottleneck theory” and the “central capacity sharing theory.” According to the bottleneck theory, dual-task interference appears when the two tasks rely on the same processor. In this theory, this processor at any time can only be occupied by one of the two tasks (Pashler, 1994a). When the first task is being processed, the second task must wait for the first one to be finished so that the processor is released. Dividing each task into three stages of (1) perceptual, (2) response selection or decision, and (3) motor execution, the bottleneck theory proposes that the stimulus perception and the motor execution stages could be performed in parallel, while the decision stage is the bottleneck that could only process the two tasks in a serial manner (McCann and Johnston, 1992; Sigman and Dehaene, 2008). Many studies have proposed evidence in favor of the bottleneck theory (Pashler and Johnston, 1989; Pashler, 1994b; Ruthruff et al., 2001; Sigman and Dehaene, 2005). This theory predicts that the dual-task interference only affects the RT of the second task and has no effect on the response of the first task because the first task is processed by the decision stage first and postpones the processing of the second task (Pashler, 1994a).

On the other hand, the central capacity-sharing theory suggests that the limitation in the processing capacity is the main reason for dual-task interference. Unlike the bottleneck theory that assumes serial processing of the two tasks, this theory suggests that in the dual-task conditions, all three stages of perceptual, decision, and motor execution could process the two tasks in parallel (Posner and Boies, 1971; Kahneman, 1973; McLeod, 1977; Duncan, 1980). In this theory, only the decision process is limited in capacity, while there are no resource limitations for the perceptual and motor execution stages (Tombu and Jolicoeur, 2003). This theory predicts that dual-task interference affects the RT of both the first and the second tasks and that the size of this reaction time change depends on the size of the sharing portion. Several studies have provided evidence in favor of the capacity sharing theory. Some have observed a robust effect of dual-task interference on the RT of both the first and the second tasks (Carrier and Pashler, 1995; Tombu and Jolicoeur, 2002; Oriet et al., 2005; Sigman and Dehaene, 2006; Zylberberg et al., 2012).

**Figure 1:**
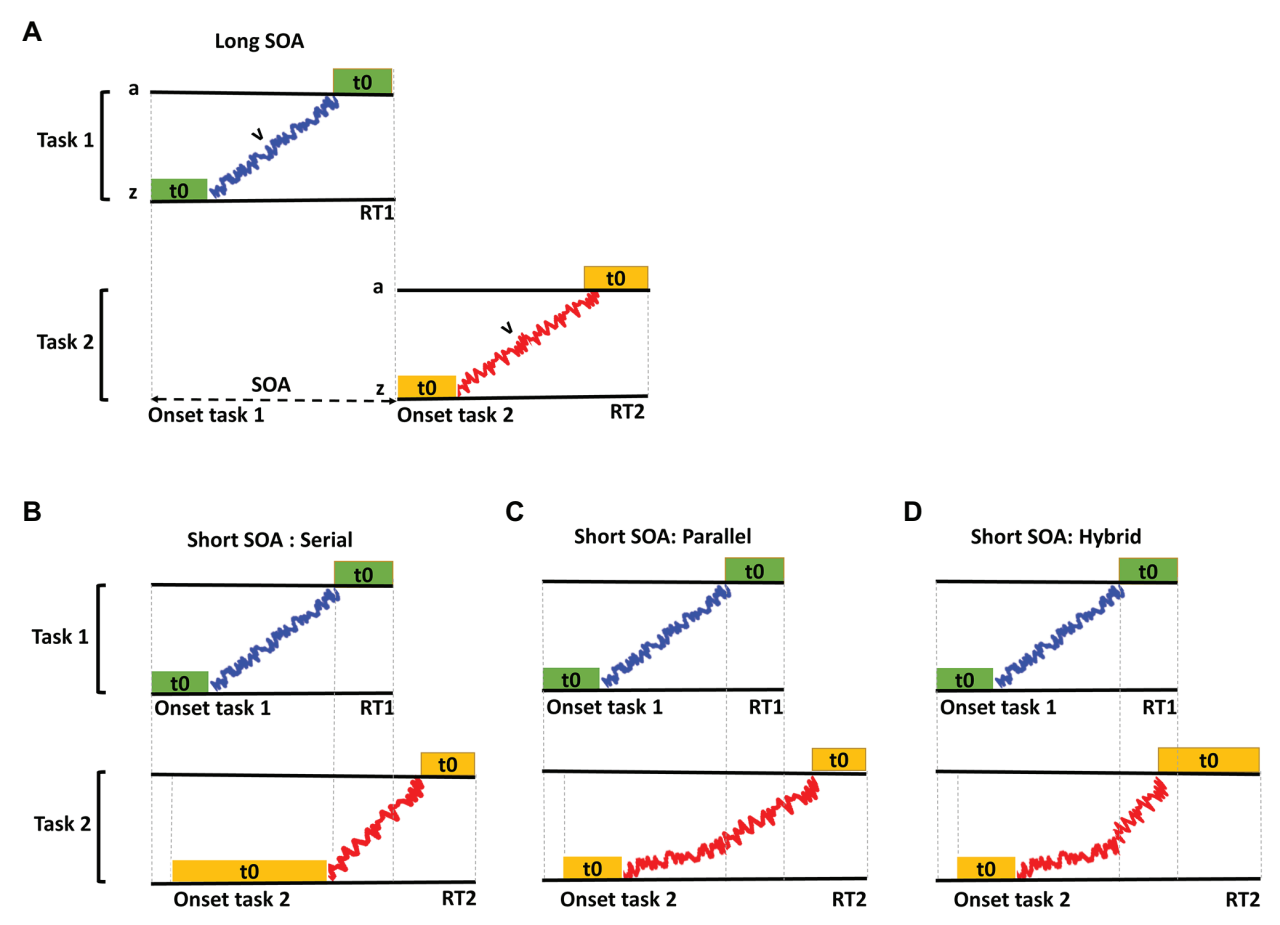
A schematic of drift-diffusion modeling based on the predictions of the bottleneck, capacity sharing theories and a recent hybrid model proposed by Zylbelberg et al. (2012). *V* denotes the noisy evidence accumulation process (drift rate) in the decision stage of the two tasks, *t0* denotes the non-decision time and *a* and *z* denote the decision threshold and the initial state of the decision processes, respectively. Here, only one threshold is shown, but there are two decision thresholds in the drift-diffusion model corresponding to the two alternatives of the two-choice tasks. **(A)** The processing stages of Task 1 (top) and Task 2 (bottom) in the long SOA condition. In the long SOA, the two tasks are processed independently, and there is no interference between the two tasks. **(B)** The processing stages of task 1 and task 2 in the short SOA based on the predictions of the bottleneck theory that suggests the evidence accumulation for Task 2 does not begin until that for Task 1 is complete. **(C)** The processing stages of Task 1 and Task 2 in the short SOA condition based on the predictions of the capacity sharing theory that suggests that the evidence accumulation for the two tasks happens simultaneously and in parallel but at slower rates compared to the long SOA conditions. **(D)** The processing stages of Task 1 and Task 2 in the short SOA condition based on the predictions of the hybrid model that suggests that the evidence accumulation for the two tasks happens simultaneously and in parallel but at slower rates and, in addition, a delay exists in the mapping of the decision to motor response in the short compared to the long SOA conditions.

Recently, Zylberberg et al. (2012) proposed a hybrid model for dual-task processing. They suggested that the decision stage of the two tasks is processed in parallel, while there exists a bottleneck in mapping the decision to the motor responses (Figure 1D). Zylberberg et al. (2012) used drift diffusion model (DDM) in a dual-task paradigm and showed that the drift rate and the post-decision time increase for the second task during dual-task interference. To do this, they used two simple artificial tasks. Currently, it is not clear whether these findings in artificial tasks could be generalized to real-world tasks such as driving. In the current study, we aimed to extend these findings to a naturalistic setting and investigate the nature of dual-task interference in our simulated driving environment. To do this, we explored the effect of SOA on driving performance and used a DDM to investigate if the driving and the secondary task are performed serially (as proposed by the central bottleneck theory) or in parallel (as proposed by the capacity sharing theory) or if a hybrid model best accounts for the results (as proposed by the Zylberberg et al., 2012).

A DDM could be used as a framework to model the different processing stages of two-choice tasks (Ratcliff, 1978, 2015; Ratcliff and Rouder, 1998). This model assumes that during a two-choice decision task, evidence accumulates gradually to reach one of two decision thresholds corresponding to the two choices. The perceptual, motor, and other non-decision related stages of task processing are modeled as the non-decision time in the DDM (henceforth referred to as non-decision time; Figure 1). The predictions of the bottleneck and the capacity sharing theories can be restated within the framework of the DDM. The bottleneck theory assumes that the decision stage of the two tasks is processed separately and sequentially and that at shorter SOAs, the processing of the decision stage of the second task is delayed until the decision stage of the first task is completed (Figure 1B). In other words, this theory predicts that the rate of evidence accumulation (drift rate) for the two tasks is constant across SOAs, while there is a delay before the start of evidence accumulation for the second task that translates to increased non-decision time at shorter SOAs. On the other hand, the capacity sharing theory suggests that the decision process for the two tasks are performed concurrently, and the resources for decision making are shared between the two tasks (Figure 1C). Therefore, this theory predicts a decrease in the rate of evidence accumulation of the two tasks at shorter SOAs and a constant non-decision time across SOAs. A hybrid account will have signatures of both bottleneck and capacity sharing theories, showing a decrease in the rate of evidence accumulation as well as an increased non-decision time.

Evidence for or against dual-task theories is mostly gathered through simple tasks. Typical examples include visual discrimination tasks (e.g., object, color, and orientation discrimination) or tone discrimination tasks (e.g., high pitch vs. low pitch). The predictions of these theories have not been sufficiently tested in more naturalistic, real-world conditions. Several differences exist between artificial tasks and real-world tasks such as driving. Examples of these include: (1) in the real-world driving situations, people often need to perform two or more motor movements sequentially to complete each driving task. For example, when the driver decides to turn right/left, he/she should rotate the wheel to turn the car to the correct location, and after a certain amount of time, turn the wheel in the opposite direction to straighten the car. This constraint may increase the demands of the driving task compared to other artificial tasks that usually require a single motor movement. (2) In the real-world driving events, time is a critical factor, and slow RTs might cause accidents. Most driving tasks have an intrinsic time limitation, while most artificial tasks do not put any constraints on the participant’s response times. This intrinsic time limitation may alter behavior in a natural setting compared to an artificial one. (3) In artificial dual-task experiments, none of the two tasks are intrinsically more important than the other one. In a dual-task paradigm, the main task is often the driving task, and the secondary task has less priority. This priority may also affect behavior in a dual-task paradigm. (4) The driving environment is a continuous environment that includes distracting elements in the scene, including the road and roadside elements, the movement in the scene caused by the interaction of the participant with the car, the car dashboard, odometer, and other car elements. These elements could alter behavior by either distracting the participants or facilitating the responses by providing an immersive experience. Most artificial tasks are discrete and contain isolated stimuli and a display that is not contingent upon the participants’ responses. Considering these factors, in the current study, we designed a dual-task paradigm in a simulated driving environment to get one step closer to the real-world dual-task conditions. Although we are aware our paradigm does not replicate real-world driving, we think it has some of the main parameters of a lane change task in a driving situation. The first goal of this study is to measure the effect of SOA on the amount of dual-task interference in this paradigm and to examine the validity of dual-task theories in more naturalistic settings.

In most dual-task studies, the order of the presentation of the tasks has been kept fixed and predictable, and participants were explicitly instructed to perform the two tasks according to the order of the presentation. In contrast, task order is often random and unpredictable in real-world situations. One open question is whether the order of the response to the two tasks during driving is specified based on a first-come, first-served basis in which the order of the presentation determines the order of response, or a higher-order control mechanism determines this order.

In dual-task studies with simple designs (Sigman and Dehaene, 2005) in which the presentation order of the tasks is kept constant, and participants are often instructed to respond to the two tasks based on the presentation order, the first-come, first-served principle usually applies. However, recent studies which have made the order of the presentation of the two tasks unpredictable and have imposed no constraints for responding to the tasks according to the presentation order, support a higher-order control mechanism for managing the timing of the response to the two tasks (Sigman and Dehaene, 2006; Szameitat et al., 2006; Huestegge and Koch, 2010; Fernández et al., 2011; Leonhard, 2011). These studies have shown that increasing the perceptual difficulty of one of the tasks, such as degrading the stimulus, causes that task to be performed second (Sigman and Dehaene, 2006; Strobach et al., 2018; but see also Leonhard, 2011 for evidence on the contrary). Similarly, an increase in the difficulty of the decision (Fernández et al., 2011) or motor execution stages (Ruiz Fernández et al., 2013), causes participants to respond to that task later. These studies suggest that participants optimize the response order to decrease the total reaction time in dual-task conditions (Miller et al., 2009). All these studies have used simple artificial tasks rather than real-world naturalistic ones. It is still an open question if a higher-order control mechanism contributes to the response order in a naturalistic setting, such as a simulated driving environment. The second goal of this study was to measure the effect of task order predictability (OP) on the responses of the two tasks and the parameters of the DDM in naturalistic settings.

In sum, we aimed to investigate the underlying mechanism of dual-task interference in a simulated driving environment using drift-diffusion modeling. The paradigm consisted of a lane change task and an image discrimination task. We investigated the effect of SOA and the predictability of the order of the two tasks on the amount of dual-task interference. Using a DDM, we investigated whether the two tasks are processed in parallel or serially and how the predictability of the order of the two tasks influenced their processing. If the decision stages of the two tasks are processed serially, as predicted by the bottleneck theory, we expect the drift rate of the second task to be independent of SOA, and the non-decision time of the second task to be dependent on SOA. In contrast, if the decision stages of the two tasks are processed in parallel according to the predictions of the capacity sharing theory, we expect the drift rate of the second task to change and the non-decision time of the second task to not change across SOAs. Finally, if the decision stages of two tasks are processed in parallel, but there is some bottleneck in the process, as predicted by the hybrid model, we expect the drift rate and non-decision time of the second task to be dependent on SOA. These results will shed light on the underlying mechanisms of dual-task interference in more naturalistic settings.

## Materials and Methods

### Participants

Twenty healthy, right-handed adults (11 females), aged 20–30, participated in the study. All participants had normal or corrected to normal vision. Additionally, all participants were not expert video game players, as defined by having less than 2 h of video-game usage per month in the past 2 years. All participants gave informed consent and were compensated for their participation.

### Stimuli and Procedure

The dual-task paradigm consisted of a lane change driving task and an image discrimination task. The driving environment was designed in the Unity 3D game engine. Participants sat at a distance of 50 cm from a 22″ LG monitor with a refresh rate of 60 Hz and a resolution of 1,920 × 1,080 and responded to the tasks using a computer keyboard.

**Figure 2:**
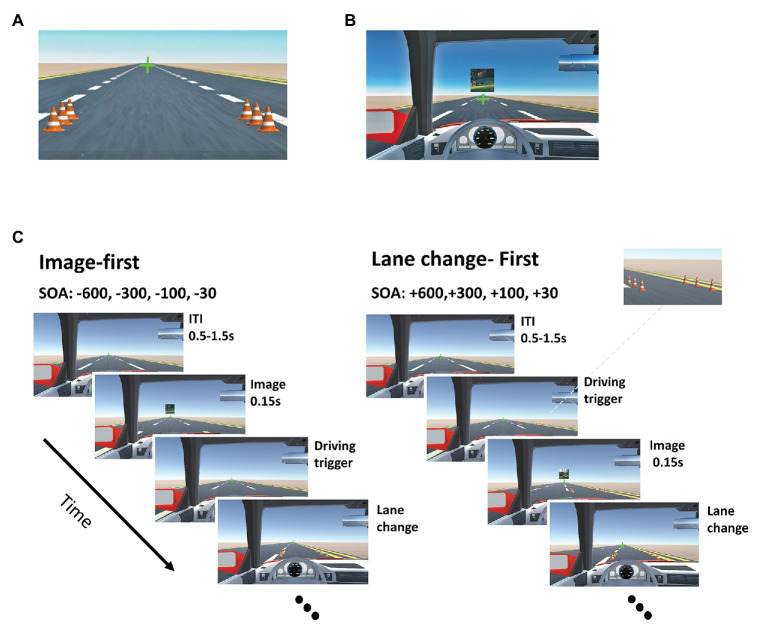
Dual-task paradigm. **(A)** A sample display showing the driving stimulus consisting of two rows of traffic cones in the middle driving lane. The cones were randomly presented in each lane, and participants had to drive through them without collision. **(B)** A sample display showing an image discrimination presented above the fixation point. Participants determined if the image was a face or a scene. **(C)** The sequence of events for a sample trial in which the image task was presented first (left), and another in which the driving task was presented first (right). The inter-trial interval (ITI) varied between 0.5 and 1.5 s. The image lasted for 150 ms, and the cones were presented 30, 100, 300, or 600 before or after the image. Participants had to perform a lane change immediately after the appearance of the cones, and an image discrimination task immediately after the presentation of the image.

The driving environment consisted of a three-lane, desert road, without left/right turns or inclining/declining hills. Driving stimuli, composed of two rows of traffic cones (three cones in each row; Figure 2A), were presented on the two sides of one of the lanes in each trial, and the participants had to immediately redirect the car to the lane with the cones and pass through the cones. The space between the two rows of cones was such that the car could easily pass through them without collision. The cones were always presented in the lanes immediately to the left or immediately to the right of the car’s lane so that the participants had to change only one lane per trial. The lane change was done gradually: the participant had to hold the corresponding key to direct the car in between the two rows of cones, and then release the key when the car was situated correctly. Any early or late key press or release would cause a collision with the cones and a performance loss in that trial. The fixation cross was jittered for 100 ms to provide online feedback in case of a collision with the traffic cones. The participants were instructed not to change lane before the cones appeared. Trials in which participants changed lane before the presentation of the cones were considered false and removed from the analysis. Using this method, we could divide a continuous driving task into individual trials with predetermined onset and ends. At the beginning of the block, participants speeded up to 80 km/h using the “up” arrow key with the middle finger of the right hand. During the block, the speed was kept constant, and the lane change was performed by pressing the right and left arrow with the middle and index fingers of their right hand, respectively. For the image discrimination task, a single image of either a scene or a face was presented for 150 milliseconds centered at 2° eccentricity above the fixation cross (Figure 2B). The size of the image was 2.5° of visual angle. Participants pressed the “*x*” and “*z*” keys on the computer keyboard with the middle and index fingers of their left hand to determine whether the image was a face or a scene, respectively. The images were pseudo-randomly selected from a set of 864 images of scenes and 435 images of faces. We selected only natural scenes and neutral faces. If participants responded incorrectly, the green fixation cross turned red, and if they responded late, it turned orange for 100 ms. The length of each trial was 3 s, and the inter-trial interval varied randomly from 0.5 to 1.5 s. For the first trial in each block, the onset of the trial was set to 2 s after the beginning of the block. The end of the trial was set to when the rear end of the car reached the end of the set of traffic cones.

The experiment consisted of two different conditions: (1) “Predictable” task order condition, and (2) “Unpredictable” task order condition. In two experimental conditions, the two tasks were presented with eight possible SOAs (−600, −300, −100, −30, +30, +100, +300 and +600 ms). In the negative SOAs, the image discrimination was presented first (image-first, Figure 2C), and in the positive SOAs, the lane change was presented first (lane change-first, Figure 2C). In the Predictable conditions, the order of the presentation was fixed, so that in two of the four blocks, the driving task was presented first, and in the other two, the image discrimination task was presented first. In the Unpredictable condition, the order of the presentation of the two tasks was not predictable in each trial. Trials with driving as the first task were interleaved with trials with the image discrimination as the first task. Before the start of each block, participants were informed about the type of the block.

In addition to the dual-task conditions, participants performed two single-task conditions: (1) single driving task and (2) single image discrimination task. In the single-task conditions, both the lane change and image stimuli were presented, but the participant only responded to one of them, ignoring the other. In the single image discrimination condition, the driving was on autopilot, and participants only responded to the images. In the single lane change condition, participants performed the lane change task and ignored the images.

Participants were told to focus on the fixation cross at the center of the page and respond to each task as fast as possible. At the end of each block, participants were informed about their performance on each task as well as their total performance. The performance in the driving task was calculated as the percentage of trials in which the participant passed through the cones without collision. The performance in the image discrimination task was calculated as the percentage of correct identifications.

Participants completed four blocks of 64 trials for each dual-task condition and two blocks of 32 trials for each single-task condition. There was a 1-min interval between blocks and a 5-min break after finishing all the blocks in each condition. The order of the blocks was counterbalanced across participants.

Before performing the main experiment, all participants performed a block of 20 trials for every single-task. If their accuracy was 80% or higher, they proceeded to the main experimental blocks. Otherwise, they repeated blocks of 40 trials for each task until they reached 80% accuracy. After the single-task training, participants performed the dual-task training block. The dual-task training was similar to the single-task training block, with the difference that if after 20 trials, the dual-task performance did not reach the 75% threshold, the training was repeated with blocks of 50 trials.

### Drift Diffusion Model Fitting

To investigate if the two tasks were processed serially, or in parallel we used a DDM in which each trial was modeled as a combination of a non-decision time and a decision time consisting of a random drift towards decision bound (Figure 1). Model parameters consisted of: (1) parameter *z* denoting the starting point of the decision process, (2) parameter *a* denoting the decision threshold, (3) parameter *v* representing the speed of information accumulation or drift rate, and (4) parameter *t0* denoting the non-decision time pertaining to the combination of all other times in the trial excluding the drift-diffusion time. The DDM was implemented in the current study, by fitting the parameters *z*, *a*, *v*, and *t0*. We modified the DDM, so that *z* and *a* were independent of SOA, and *v* and *t0* were dependent on SOA. Therefore, in the modified DDM, four values were fit for the parameter *v* and four values for the parameter *t0* corresponding to the four SOAs, one value for the parameter *a* and one value for the parameter *z* across all SOAs.

We used the Fast-dm package, developed by Voss and Voss (2007), for model fitting. Fast-dm is a package for fast drift-diffusion modeling. This package uses a partial differential equation method and a simplex routine to obtain the parameters of the DDM, and uses the calculated cumulative density function (CDF) of the predicted RTs to estimate the goodness of fit using a Kolmogorov-Smirnov (KS) function (Voss and Voss, 2008; Voss et al., 2015). The DDM was fit separately for each task (lane change/image discrimination task) and each participant. We also calculated *R*^2^ values as an additional measure to examine the goodness of fit of the model.

### Data Analysis

Only the correct trials were used for the RT analysis. In the dual-task conditions, if the response to both tasks was correct, that trial was included in the analysis. The trials in which the reaction time to each of the tasks was <200 ms and >1,500 ms were excluded from the analysis (3.48% of the trials). To quantify the effect of SOA on RTs and DDM parameters, one-way repeated-measures ANOVAs were used and to quantify the effect of SOA and task conditions on RTs, accuracies, and DDM parameters, two-way repeated-measures ANOVAs were used. A Greenhouse-Geisser correction was performed when sphericity had been violated. To compare the threshold, slope, and shift of the logistic regression function between the two task conditions, a paired t-test was used. We also performed three-way repeated measure ANOVAs with task condition, task order and SOA as three factors. The details of the statistical results are placed in Supplementary Tables S1–S3. In addition, we used t-test to statistically compare RTs, accuracies and DDM parameters between task conditions (dual vs. single/predictable vs. unpredictable) for each SOA. The details of the statistical tests for this analysis are placed in Supplementary Tables S7–S9. False Discovery Rate correction (Benjamini and Hochberg, 1995) was applied in all cases that multiple comparisons were performed.

We used a logistic regression model to examine the effect of SOA and OP on the order of the response of the two tasks. The probability that the lane change response was initiated before the image discrimination response was determined by the following formula:

$$Logit\;\;\;[P]\;\;\;=\;\;\;\beta_{0}\;\;+\;\;\;\beta_{1}C$$

where P stands for the probability that the lane change task was responded to first and C stands for SOAs. Parameters *β*_0_ and *β*_1_ were calculated for each participant. The model was fit separately on the data from the two dual-task conditions. A maximum likelihood estimation procedure was used for curve fitting.

## Results

### Effect of Dual-Task Interference on RTs

**Figure 3:**
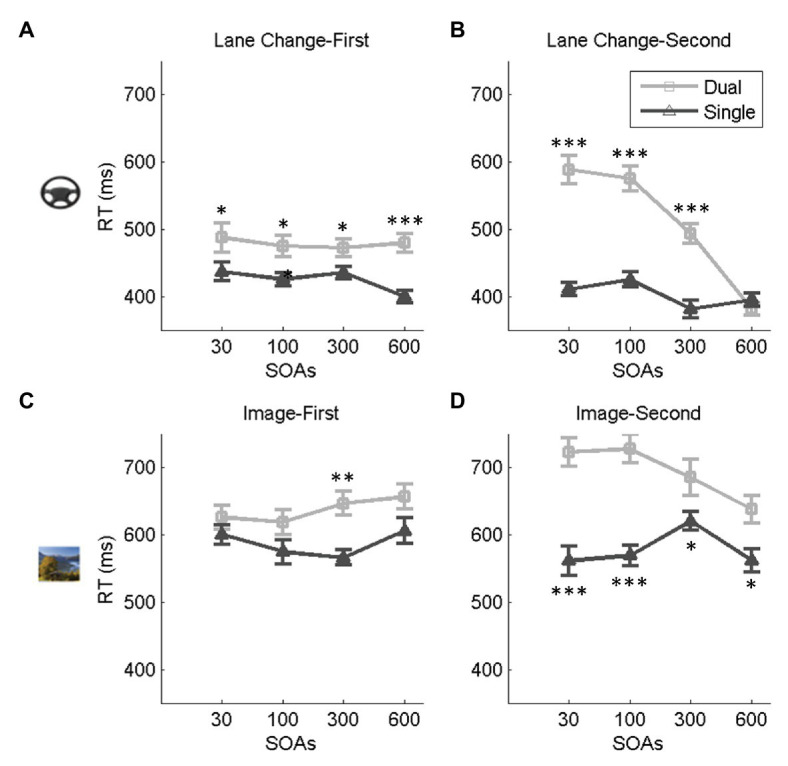
Effect of task condition (dual vs. single) and SOA on RTs. **(A,B)** These panels indicate the RTs for the lane change in the lane change-first and lane change-second task orders, respectively, for the single-task (red) and the dual-task (blue) conditions. **(C,D)** These panels show the image discrimination RTs in the single (red) and the dual (blue) task conditions for the image-first and the image-second task orders, respectively. In all panels, errorbars show standard errors of mean and stars show a significant difference between task conditions for each SOA (^*^ < 0.05, ^**^ < 0.01, and ^***^ < 0.001).

We first focused our analysis on the dual-task condition with the predictable task order and compared it with the single-task conditions (Figure 3). We ran four two-way repeated-measures ANOVAs with task condition (dual/single), and SOA as factors separately for the lane change and the image discrimination and the lane change-first and image-first task orders. Table 1 contains the details of the statistical results. Results showed a significant main effect of task condition with longer RTs in the dual‐ compared to the single-task condition in all cases [*Fs*(1,19) > 6.21, *p*s < 0.023, $\;\eta_{p}^{2}$ > 0.24]. The effect of SOA was significant in all cases [*Fs*(3,57) > 6.5, *p*s < 0.006, $\eta_{p}^{2}$ > 0.25] except for the lane change RTs in the lane change-first task order [*F*(1.49, 26.84) = 2.55, *p* = 0.099, $\eta_{p}^{2}$ = 0.11]. The interaction between task condition and SOA was also significant in all cases [*Fs*(1,57) > 3.05, *p*s < 0.041, $\eta_{p}^{2}$ > 0.13]. Further comparisons looking at the effect of SOA on RTs in the dual-task condition using one-way repeated-measures ANOVAs showed a significant effect of SOA on the RTs in all cases [*Fs*(3,57) > 3.95, *p*s < 0.015, $\eta_{p}^{2}$ > 0.17] except for the lane change when it was presented first [*F*(1.6, 28.95) = 2.55, *p* = 0.49, $\eta_{p}^{2}$ = 0.02]. Consistent with previous studies of dual-task interference (Pashler and Johnston, 1989; Tombu and Jolicoeur, 2002; Sigman and Dehaene, 2005), when the image discrimination or the lane change tasks were presented second, the RTs increased at shorter SOAs. Interestingly, when the image discrimination was presented first, decreasing SOAs had an opposite effect, with shorter SOAs showing faster RTs. These results have not been observed in previous dual-task studies and might be driven by participant’s urge to finish the image discrimination task sooner in order to reduce the interference on driving.

**Table 1:** Results of two-way repeated-measure ANOVAs for the effect of task condition (dual vs. single), SOA, the interaction between the two on RTs, and SOA in dual shows the results of one-way repeated-measures ANOVAs for the effect of SOA on RTs separately in the dual-task condition.

		Lane change-first	Lane change-second	Image-first	Image-second
Task condition (dual vs. single)	*F*	10.19	43.10	6.22	23.20
	*df*	1, 19	1, 19	1, 19	1, 19
	*p*	**0.005**	**0.002**	**0.022**	**<0.0001**
	$\eta_{p}^{2}$	0.349	0.694	0.247	0.550
SOA	*F*	2.55	101.60	7.29	6.56
	*df*	1.49, 26^*^	3, 57	3, 57	1.8, 39^*^
	*p*	0.099	**0.0002**	**0.0002**	**0.005**
	$\eta_{p}^{2}$	0.119	0.843	0.277	0.257
Task condition × SOA	*F*	3.05	48.24	3.24	6.99
	*df*	3, 54	1.57, 29^*^	3, 57	3, 57
	*p*	**0.036**	**0.002**	**0.041**	**0.002**
	$\eta_{p}^{2}$	0.138	0.717	0.146	0.269
SOA in dual	*F*	0.657	103.7	7.10	8.35
	*df*	1.60, 28^*^	1.43, 27^*^	3, 57	1.61, 31^*^
	*p*	0.491	**0.002**	**0.002**	**0.002**
	$\eta_{p}^{2}$	0.023	0.845	0.272	0.305

All *p*-values were corrected for multiple comparisons, and Greenhouse-Geisser correction was done when necessary (indicated by a star). The significant p-values were shown in bold.

**Table 2:** One-way repeated-measures ANOVAs for the effect of SOA on *v* and *t0*.

		Lane change-first		Lane change-second		Image-first		Image-second	
		*v*	*t0*	*v*	*t0*	*v*	*t0*	*v*	*t0*
SOA	*F*	1.59	3.46	15.03	64.42	3.95	7.96	6.67	12.20
	*df*	1.87, 35.53^*^	1.60, 30.43^*^	3, 57	1.59, 30.37^*^	3, 57	1.35, 25.80^*^	3, 57	1.86, 35.36^*^
	*p*	0.217	0.663	**0.002**	**0.001**	**0.022**	**0.006**	**0.002**	**0.001**
	$\eta_{p}^{2}$	0.078	0.018	0.442	0.772	0.172	0.295	0.296	0.391

All *p*-values were corrected for multiple comparisons and Greenhouse-Geisser correction was done when necessary (indicated by a star). The significant p-values are shown in bold.

Further analysis showed that the image discrimination RTs were generally longer than the lane change task RTs (Supplementary Figure S3), but the magnitude of the dual-task effect was not different between the tasks (for more details see Supplementary Table S6). We also investigated if the image type (scenes vs. faces) affected RTs. Results showed no significant difference between scene image RTs and face image RTs [*t*(159) = 1.13, *p* = 0.11]. Also, the lance change RTs did not change in trials in which the image was a scene compared to those in which it was a face [*t*(159) = 1.57, *p* = 0.118].

We also calculated the accuracy of participants in single‐ and dual-task conditions. Results showed that the accuracies were above 95 and 90% for all conditions of the lane change task and the image discrimination task, respectively (Supplementary Figures S1, S2).

In sum, our results show a clear effect of SOA on driving and image discrimination RTs. The presence of these strong effects allows us to use SOA as a factor for drift-diffusion modeling in the next section to investigate the nature of dual-task interference in our simulated driving set up.

### Drift-Diffusion Modeling of the Effect of Dual-Task Interference on RTs

Drift diffusion modeling was used to investigate if a change in SOA affects the drift rate, non-decision time, or both. The model could account for most of the variance in the data (*R*^2^: Lane change-first 0.78 ± 0.03, Lane change-second 0.94 ± 0.02, Image-first 0.71 ± 0.04, and Image-second 0.84 ± 0.03), and the distribution of the RTs from the model fit was not significantly different from the original data in all subjects and all conditions (*ps* > 0.1).

**Figure 4:**
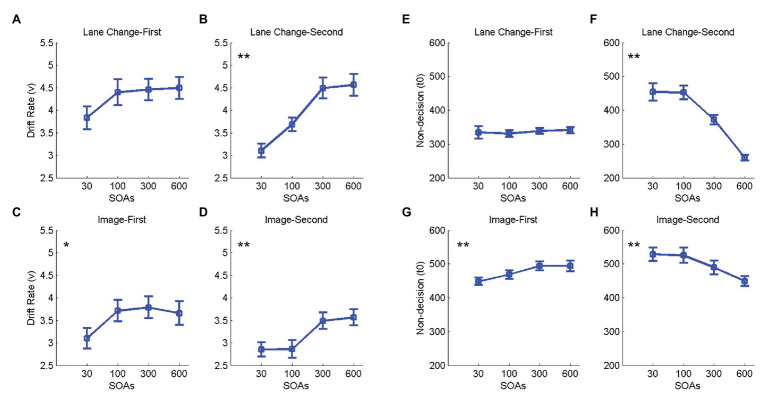
Effect of SOA on drift rates (*v*) and non-decision times (*t0*). Panels **(A–D)** on the left show the effect of SOA on the drift rate (*v*) for lane change in the lane change-first **(A)** and lane change-second **(B)** conditions and that for the image discrimination in the image-first **(C)**, and image-second **(D)** conditions. Panels **(E–H)** on the right show the effect of SOA on non-decision time (*t0*) for lane change task in the lane change-first **(E)** and lane change-second **(F)** conditions and that for the image discrimination task in the image-first **(G)** and image-second **(H)** conditions. In all panels, errorbars show standard errors of mean and stars show a significant effect of SOA (^*^ < 0.05 and ^**^ < 0.01).

Next, we investigated the effect of SOA on the two model parameters *v* and *t0*, corresponding to the drift rate and non-decision times. Serial processing of the two tasks would lead to an increase in the *t0* for the second task, while parallel processing of the two tasks would decrease the *v* for the second task at shorter SOAs. Results showed that when either of the two tasks was presented second, *v* decreased and *t0* increased at shorter SOAs [*Fs*(3,57) > 6.66, *ps* < 0.003, $\eta_{p}^{2}$ > 0.29; Figures 4B,D,F,H]. No significant change in *v* or *t0* was observed when driving was presented first (*p* > 0.05; Figures 4A,E) and a decrease in both *t0* and *v* was observed at shorter SOAs when the image discrimination was presented first [*Fs*(3,57) > 3.94, *ps* < 0.023, $\eta_{p}^{2}$ > 0.17; Figures 4C,G]. The details of statistical tests are shown in Table 2. These results suggest that the two tasks are neither processed in a strictly parallel nor a strictly serial manner, as a change in the non-decision time is always accompanied by a change in the drift rate.

**Table 3:** Results of two-way repeated-measures ANOVAs for the effect of OP and SOA on RTs and one-way repeated-measures ANOVAs for effect SOA on RTs in the unpredictable condition.

		Lane change-first	Lane change-second	Image-first	Image-second
Task condition (predictable vs. unpredictable)	*F*	10.21	11.81	3.53	4.29
	*df*	1, 19	1, 19	1, 19	1, 19
	*p*	**0.005**	**0.012**	0.076	0.069
	$\eta_{p}^{2}$	0.350	0.383	0.157	0.184
SOA	*F*	2.83	88.89	3.35	7.55
	*df*	1.48, 28^*^	1.51, 28^*^	3, 57	1.65, 31^*^
	*p*	0.089	**0.0004**	**0.050**	**0.006**
	$\eta_{p}^{2}$	0.130	0.824	0.150	0.285
Task condition × SOA	*F*	5.02	7.06	3.37	3.07
	*df*	3, 57	3, 57	3, 57	1.83, 34^*^
	*p*	**0.003**	**0.004**	**0.041**	0.057
	$\eta_{p}^{2}$	0.215	0.271	0.151	0.139

All *p*-values were corrected for multiple comparison and Greenhouse-Geisser correction was done when necessary (indicated by a star). The significant p-values are shown in bold.

### Effect of Task OP on RTs

**Figure 5:**
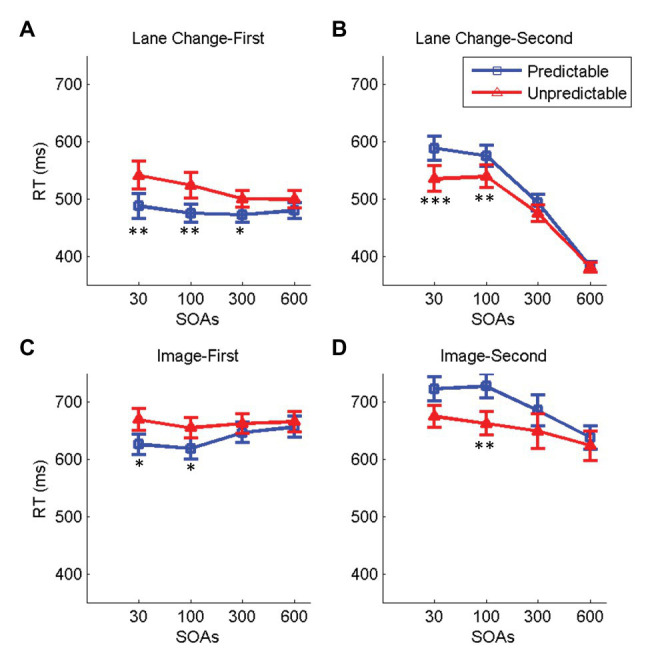
Effect of OP and SOA on RTs. The two top panels show the RTs for the lane change in the lane change-first **(A)** and lane change-second **(B)** task orders for the predictable (blue) and the unpredictable (red) task order conditions. The two bottom panels show the RTs for the image discrimination task in the image-first **(C)** and image-second **(D)** task orders for the predictable (blue) and the unpredictable (red) task order conditions In all panels, error bars show standard errors of mean and stars show a significant difference between task conditions for each SOA (^*^ < 0.05, ^**^ < 0.01, and *** < 0.005).

To investigate the effect of task OP on the RTs during dual-task performance, we compared the main dual-task condition in which the task orders were predictable (i.e., the two task orders were presented in separate blocks) to a condition in which the task orders were unpredictable and varied randomly from trial to trial within a block. We ran four two-way repeated-measures ANOVAs with task condition (predictable/unpredictable) and SOA as the two factors, separately for the lane change and the image discrimination, and the lane change-first and image-first task orders. The details of the statistical tests are summarized in Table 3. The effects of OP, SOA, and their interaction on RTs were significant in both lane change-first and lane change-second conditions [*Fs* > 5.03, *ps* < 0.013, $\eta_{p}^{2}$ > 0.21; Figures 5A,B] except for the effect SOA on the lane change-first that was marginally significant [*F*(1.48,28) = 2.83, p = 0.089, $\eta_{p}^{2}$ = 0.13]. When the image discrimination was presented first (Figure 5C), OP had a marginally significant effect on mean image discrimination RTs [*F*(1,19) = 3.54, *ps* = 0.076, $\eta_{p}^{2}$ = 0.15], and the interaction between OP and SOA was significant [*F*(3,57) = 3.37, *ps* < 0.041, $\eta_{p}^{2}$ > 0.15]. When the image discrimination was presented second (Figure 5D), the effect of OP on RTs [*F*(1,19) = 4.29, *ps* = 0.069, $\eta_{p}^{2}$ = 0.18], and the interaction between OP and SOA on RTs [*F*(3,57) = 3.07, *ps* = 0.057, $\eta_{p}^{2}$ = 0.13] were marginally significant.

**Table 4:** Results of two-way repeated-measures ANOVAs for the effect of OP and SOA on *v* and *t0*.

		Lane change-first		Lane change-second		Image-first		Image-second	
		*v*	*t0*	*v*	*t0*	*v*	*t0*	*v*	*t0*
OP	*F*	4.02	3.06	0.283	11.27	0.891	0.019	0.002	5.07
	*df*	1, 19	1, 19	1, 19	1, 19	1, 19	1, 19	1, 19	1, 19
	*p*	0.236	0.128	0.801	**0.012**	0.714	0.893	0.968	**0.052**
	$\eta_{p}^{2}$	0.175	0.139	0.015	0.372	0.045	0.001	0.001	0.211
SOA	*F*	2.10	1.33	28.02	43.23	7.23	9.03	3.45	5.93
	*df*	1.74, 33^*^	1.44, 27^*^	3, 57	1.40, 26^*^	3, 57	1.73, 32^*^	3, 57	1.93, 36^*^
	*p*	0.143	0.319	**0.002**	**0.002**	**0.002**	**0.002**	**0.036**	**0.008**
	$\eta_{p}^{2}$	0.100	0.056	0.596	0.695	0.276	0.322	0.157	0.238
OP × SOA	*F*	1.78	3.21	1.37	10.91	2.16	1.84	3.50	1.62
	*df*	3, 57	1.87, 35^*^	2.09, 39^*^	3, 57	3, 57	2.07, 39^*^	3, 57	2.19, 41^*^
	*p*	0.188	0.110	0.188	**0.003**	0.188	0.208	0.112	0.208
	$\eta_{p}^{2}$	0.086	0.145	0.084	0.365	0.100	0.089	0.156	0.079

All *p*-values were corrected for multiple comparisons and Greenhouse-Geisser correction was done when necessary (indicated by a star). The significant p-values are shown in bold.

Furthermore, we investigated the effect of SOA separately in the unpredictable conditions using one-way repeated-measures ANOVAs (note that the effects for the predictable condition are already reported in the previous section). The results showed a significant effect of SOA on the RTs in all cases [*Fs*(3,57) > 3.75, *ps* < 0.015, $\eta_{p}^{2}$ > 0.16] except for when the image discrimination was presented first [*Fs*(3,57) = 0.62, *ps* < 0.52, $\eta_{p}^{2}$ > 0.03].

In general, these results demonstrate that OP increases the mean RT of the first task and decreases the mean RT of the second task with the changes more pronounced when the tasks get closer together in time. These results show that unpredictability of the task order attenuates the effect of SOA on RTs for all cases except the lane change-first RTs. We next investigated the possible origin of this attenuation effect.

### Effect of Task OP on the Response Order

**Figure 6:**
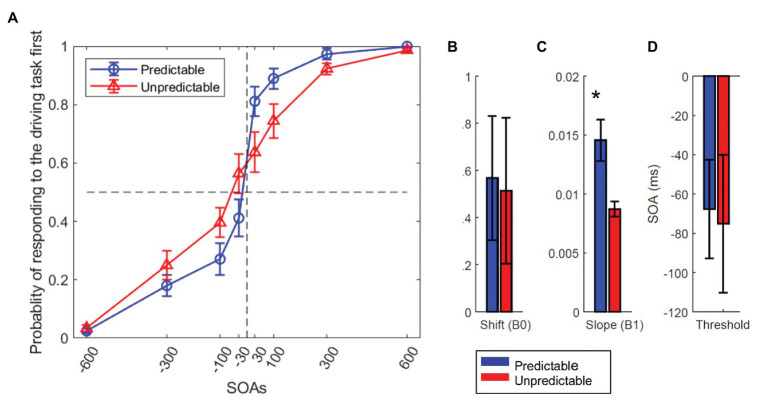
Effect of OP on the response order. Predictable and Unpredictable conditions are shown in blue and red colors, respectively. **(A)** The probability of first responding to the lane change task plotted for the two task conditions. The curves are fit to the average data using a logistic regression function. **(B)** The shift of the logistic regression function (*β*_0_), **(C)** the slope of the logistic function (*β*_1_), and **(D)** The T50 (the SOA in which participants responded to the lane change task first with 50% probability), for the predictable (blue) and unpredictable (red) conditions. The shift did not differ between the two conditions, but the slope was shallower in the unpredictable condition (*p* < 0.006). There was a general bias for responding to the lane change task first in both conditions. In all panels, error bars show standard errors of mean. The star shows a significant difference between task conditions (^*^ < 0.05).

To investigate the effect of SOA and OP on the order of the response to the two tasks, we calculated the probability that the lane change task was responded to first in each SOA and for each subject (Figure 6A) and fit a logistic regression model to these probability values. The model was fit separately for each of the two dual-task conditions, and an intercept (*β*_0_ in the logistic model described in the methods) and a slope (*β*_1_ in the logistic model) was calculated for each condition and each participant. We also calculated the SOA value in which the probability of responding to the lane change task first was 50% (T50). Then, to quantify the effect of OP on the response order, the model outputs and the T50 value across the two experimental conditions were submitted to a paired t-test. OP had no significant effect on the shift (*β*_1_) of the logistic function [*t*(1,19) = 0.323 *p* = 0.75; Figure 6B]. The slope of the logistic function (*β*_1_) was significantly influenced by OP [*t*(1,19) = 3.08, *p* = 0.006]. Negative T50 values in both conditions show that participants had a general bias to respond to the lane change task first (Figure 6C) but this bias was the same across the two conditions [*t*(1, 19) = 0.317, *p* = 0.75]. At SOA = 0, in more than 60% of trials lane change was responded to first. In sum, these results showed that OP changes the response order to the two tasks and has no effect on the bias in favor of the lane change task.

### Drift-Diffusion Modeling of the Effect of Task OP on RTs

**Figure 7:**
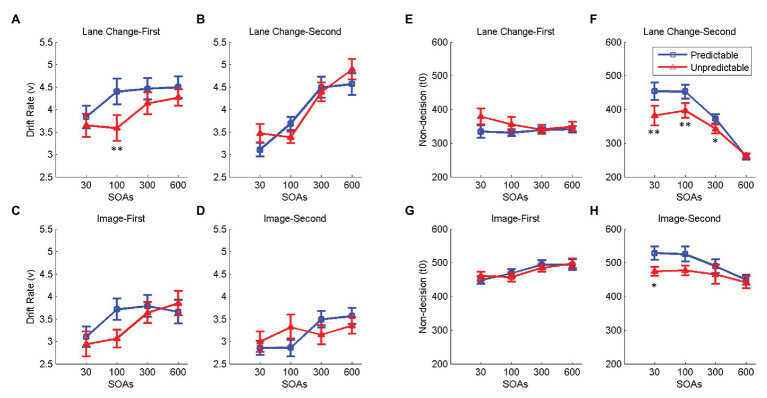
Effect of OP and SOA on drift rates (*v*) and non-decision times (*t0*). Blue lines and red lines show the predictable and unpredictable task orders, respectively. Panels **(A–D)** on the left show the effect of OP and SOA on the drift rate (*v*) for lane change in the lane change-first **(A)** and lane change-second **(B)** conditions and that for the image discrimination in the image-first **(C)** and image-second **(D)** conditions. Panels **(E–H)** on the right show the effect of OP and SOA on non-decision time (*t*0) for lane change in the lane change-first **(E)** and lane change-second **(F)** conditions and that for the image discrimination task in the image-first **(G)** and image-second **(H)** conditions. In all panels, errorbars show standard errors of mean and stars show a significant difference between task conditions for each SOA (^*^ < 0.05 and ^**^ < 0.01).

Drift Diffusion Model (DDM) was fit to the data from the predictable and unpredictable task order conditions, separately, and output model parameters were compared for the two conditions. The results of model fitting on the unpredictable task order condition showed that the model could account for most of the variance in the data (*R*^2^: lane change-first 0.70 ± 0.04, lane change-second 0.96 ± 0.01, image-first 0.75 ± 0.03 and image-second 0.82 ± 0.03) and the distribution of the RTs from the model fit was not significantly different from that of the original data in all subjects and all conditions (*ps* > 0.09). We ran two-way repeated-measures ANOVAs to investigate the effect of task condition (Predictable vs. Unpredictable) and SOA on the two parameters *t0* and *v*, separately for the two task orders, and the lane change and the image discrimination tasks. The details of the statistical test are shown in Table 4. The effect of OP on *v* was not significant in all cases (*ps* > 0.05; Figures 7A–D). This effect on *t0* was only significant in the lane change-second [*F*(1,19) = 11.27, *p* = 0.012, $\eta_{p}^{2}$ = 0.37; Figure 7F] and marginally significant for image-second conditions [*F*(1,19) = 5.07, *p* = 0.052, $\eta_{p}^{2}$ = 0.21; Figure 7H] and was not significant in the lane change-first and image-first conditions (*ps* > 0.05; Figures 7E,G). SOA had a significant effect on *v* and *t0* in all conditions [*F*(3,57) > 3.54, *p* < 0.02, $\eta_{p}^{2}$ > 0.15], except when the lane change task was presented first (*p* > 0.05; Figure 7A). The interaction of OP and SOA on *t0* was only significant for lane change-second conditions [*F*(3,57) = 10.91, *p* = 0.003, $\eta_{p}^{2}$ = 0.36; Figure 7F]. These results show that when either the image discrimination or the lane change tasks were presented second, unpredictability changed the non-decision time of the tasks. Note that the analysis of the response order showed that in the unpredictable condition, the second task was more likely to be responded to first. The changes in the order of response could be tightly related to the decrease in the non-decision time of the second task.

## Discussion

The purpose of this study was to investigate the underlying mechanisms of dual-task interference in a simulated lane change environment. We used a systematically controlled dual-task paradigm in which an image task was presented at set times before or after the lane change task. We investigated the effect of dual-task, SOA, and unpredictability of task order on subjects’ performance and modeled the results using a DDM. Results showed strong dual-task effects on both tasks with stronger effects at shorter SOAs for the second tasks. DDM showed a change in both the drift rate and non-decision times, suggesting that a hybrid model containing features of both serial and parallel processing best accounts for the results. Unpredictability of the task order attenuated the effect of SOA by changing the order of the response to the two tasks. This effect induced a change in the non-decision time of the second task in the DDM.

The observation of a strong dual-task effect on both image discrimination and lane change RTs when they were presented second is compatible with the predictions of both capacity sharing and bottleneck theories. But our behavioral results are not fully compatible with either of the two theories. We observed a clear dual-task effect comparing the RTs of the single-task with the dual-task conditions when the tasks were presented first with longer RTs for the single-compared to the dual-task condition, and a decrease of RT at shorter SOAs for the image task. The bottleneck theory predicts no change in the RT in the single-compared to the dual-task, and the capacity sharing theory predicts an RT effect that increases at shorter SOAs. Our observations are different from those reported by Levy et al. (2006) and Hibberd et al. (2013) who only observed a dual-task effect for the second task in a simulated driving environment. In these two studies, for the driving task, participants performed a car following in which they pressed the brake pedal when the color of the brake light changed. In our study, for performing the driving, participants had to press a key, hold it, and tune the location of the car to avoid the collision. The more continuous and multi-step nature of the response in our study might have increased the time pressure and demands of the driving task, imposing a priority for processing it. This increased priority, in turn, may have caused the participant to not invest all their resources on the image task when they knew that a driving trigger may be presented soon. The increased priority may have also caused participants to try to respond to the first-presented image task faster at shorter SOAs to release resources for the driving task. These effects clearly suggest a more complex management of resources than what is suggested by the bottleneck or capacity sharing theories.

Results of our DDM analysis further confirm that neither the bottleneck theory with its prediction of a strictly serial processing of tasks nor the capacity sharing theory with predictions of a fully parallel processing can account for our results. This is because both the drift rate and the non-decision times of the second tasks are found to be modulated across SOAs. This result suggests some degree of capacity sharing for the processing of the two tasks. In addition, they suggest some delay in the processing of the second task due to a potential bottleneck. In other words, our results suggest that the best model to account for dual-task interference in driving is a hybrid model combining the two extremes suggested by capacity sharing and bottleneck theories. Zylberberg et al. (2012) modeled RT of the second task with a term for accumulation time that includes the time from stimulus onset to end of the decision process and a term for the post-accumulation time that includes the time from the end of the decision process to the motor response. Their results showed that both accumulation and post-accumulation times increase in short compared to the long SOA conditions. These results are compatible with a hybrid model as they show that the dual-task interference decreases the efficacy of evidence accumulation without halting it and causes a delay in mapping the decision to motor response. In line with Zylberberg et al. (2012), the result of the current study demonstrates that the decision stage of the second task is processed in parallel with the decision stage of the first task, and there is also some bottleneck in processing the second task. In our DDM modeling, it is not possible to determine if the bottleneck is before or after the evidence accumulation stage. It is plausible that the mapping of the decision to the motor output happens in a serial manner, and this imposes a bottleneck on the production of the response, but proof of this point requires further studies.

Results of our task order predictability manipulation suggest the involvement of an active higher order control mechanism for scheduling the tasks (De Jong, 1995; Luria and Meiran, 2003; Sigman and Dehaene, 2006; Szameitat et al., 2006; Fernández et al., 2011; Leonhard, 2011; Ruiz Fernández et al., 2013) as opposed to passive scheduling of the tasks on a first-come first-served basis (Pashler, 1994b; Bunge et al., 2000; Jiang, 2004). A passive scheduling account would predict no effect of task order predictability on response orders while in our experiment, task order predictability changed the order of the response to the two tasks causing the RTs for the first task to increase and those for the second task to decrease. Our results do not fully replicate previous studies of task order predictability in simple artificial dual-tasks (Sigman and Dehaene, 2006; Töllner et al., 2012). These studies report an increase in RT for both the first and second tasks. However, unlike our paradigm these studies have instructed the participants to respond to the stimuli according to the presentation order. Imposing this artificial response order may have increased the dual-task costs leading to longer RTs (Strobach et al., 2018). Our paradigm is closer to naturalistic settings in which the secondary task can happen at any time relative to the driving event and are more applicable to natural settings.

Another feature of our data also favors an active account of task scheduling. Participants had an overall bias to respond to the lane change task first. Order predictability had no effect on this average bias. This bias might be due to the context of the lane change task and the intrinsic time pressure for responding to the lane change task in order to avoid collision with the cone obstacles. It may also be related to the differences in the difficulties between the image and lane change tasks. Miller et al. (2009) have suggested an RT optimization model for scheduling of tasks in dual-task paradigm. This model suggests that the participants’ aim in a dual-task paradigm is to decrease the total RT (RT of the first task + RT of the second task). Therefore, they tend to respond to the easy task sooner than the difficult one. In other words, the duration of the components of the two tasks determines which task is responded to first (see Sigman and Dehaene, 2006 and Fernández et al., 2011 and Ruiz Fernández et al., 2013 for evidence in favor of this model). It is hard to evaluate if our results favor this model or not. In our paradigm, the decision time and non-decision times of the images task were slightly longer, while the motor stage of the lane change task was likely more difficult as it involved a series of motor movements. It is hard to speculate about the effect of each of these stages on the decision for task order without further experiments manipulating each stage in isolation. Regardless of the underlying reason, the prioritization of the lane change task over the image task shows that the order of the presentation of the tasks does not dictate the order of the processing.

Studies of working memory have categorized executive functions into distinct components (Jonides et al., 2008; Nee et al., 2013). These include shifting attention between items in working memory, updating the actively maintained items, and preventing interference from outside distractors and internal intrusions (Courtney et al., 2007; Bledowski et al., 2009; Nee et al., 2013). We did not have an explicit working memory task, but our behavioral and modeling results, in line with previous findings (De Jong, 1995; Meyer and Kieras, 1997; Szameitat et al., 2002; Piai and Roelofs, 2013) suggest that similar executive functions may be at play in our dual-task paradigm to coordinate which task should be prioritized and processed first, to divide the resources during the evidence accumulation of the two tasks, and to maintain the information of one task during the (possibly post-accumulation) bottleneck until the process of the other task is completed. Based on our results, we can speculate that resources are divided between tasks with a general preference for the first task and an additional preference for the lane change task. The information is then updated and maintained for the two tasks during the evidence accumulation and response selection phases, with the first task imposing constraints and interfering with the process of the second task.

We have used the broad term of interference for the phenomenon of performance decline and changes in the parameter of DDM in our dual-task paradigm. This term has been used in the literature to describe multiple distinct phenomena (Pashler, 1994a; Luck, 1998; Marois and Ivanoff, 2005; Johnston and McCann, 2006; Tombu et al., 2011), including performance declines due to internal processes and those related to distractions from external stimuli. In a dual-task paradigm, when the first task is being processed, the presence of the stimulus of the second task could serve as an external distractor. Once the process of the second task starts, the information from the second task is no longer an external distractor. The effect of this external distraction can be observed in our control single-task conditions, as in this condition, the stimuli for the ignored task are still present. Small modulations in the RTs in the single-task condition are possibly related to external distraction from the ignored task. The dual-task effect, however, is much stronger than this small modulation. This dual-task effect, observed especially in the second task, is due to proactive interference (Jonides and Nee, 2006) from the internal processing of the first task imposing a reduction in the drift-diffusion rates of the second task. Other than this interference, task shifting may play some roles in our increased RTs. As discussed above, the changes in the non-decision time could be related to the shift between the two tasks during the post-accumulation phase (Zylberberg et al., 2012).

Our simulated driving paradigm was close to a real-life driving task in some respects such as having a continuous driving scene with a multi-lane road and a car dashboard and requiring a two-step response (pressing and releasing the button at prompt times) with an intrinsic time pressure for the driving task. But our paradigm also kept the driving task and driving environment as simple as possible to control the main experiment variables systematically. Participants drove at a constant speed in a high-way desert with no hill or turn, and other cars in our paradigm. The display was viewed on a 2D computer screen as opposed to a 3D environment. The responses were collected using button presses. Participants were only focused on the lane change in the driving task as opposed to real-world settings in which the driver has to control the brake, gas pedal, and steering wheel at the same time. Lastly, our image discrimination task was not a natural secondary task (although one could argue that many real-world tasks such as identifying images on traffic signs or billboards or determining if an item by the roadside is a human or an inanimate object involve similar mechanisms as our image discrimination task). These factors limit the generalizability of our task to a real-world driving scenario. Future studies with even more realistic driving simulators could determine if our results can be translated to real-world driving.

Another factor worth considering in future studies is the gaze behavior of participants during dual-task interference. In our experiment, we asked participants to fixate on a fixation point at the center of the screen close to the focus of the radial optical flow pattern, which is the natural position of the gaze during driving (Lappe et al., 2000). As such it is likely that our participants have kept their eyes on the fixation point. However, since we did not have eye tracking in our experiment, we cannot be certain about the gaze behavior of our participants. Future studies could shed light on the gaze behavior and its potential effects on dual-task interference in a driving task.

To sum up, here, for the first time, we used a simulated driving environment and a DDM to explore the processing of two tasks in a naturalistic dual-task setting. Our finding revealed that performing a secondary task while driving deteriorates the driving performance, whether presented before or after the driving task. Further investigations showed this effect might be caused by slower parallel processing of the driving task in the presence of a secondary task with some delays in the process, suggesting that a hybrid model best accounts for the results. Our results could be applicable for optimizing the design of driving assistance systems such as road signs, alarm systems, and other driver interfaces to reduce accidents. They could also inform precautionary measures aimed at reducing accidents in clinical populations with impaired executive control and should be considered in future neuroscience studies aiming to explore the neural underpinnings of dual-task interference in natural settings.

## Data Availability Statement

The raw data supporting the conclusions of this article will be made available by the authors, without undue reservation upon request.

## Ethics Statement

The studies involving human participants were reviewed and approved by the ethics committee of Iran University of Medical Sciences (ethics code: IR.IUMS.REC.1396.0435). The patients/participants provided their written informed consent to participate in this study.

## Author Contributions

MAZ: Conceptualization, methodology, investigation, formal analysis, and writing of the original draft. GHZ: Supervision and writing ‐ review and editing. MVP: Supervision, conceptualization, methodology, and writing ‐ review and editing. All authors contributed to the article and approved the submitted version.

### Conflict of Interest

The authors declare that the research was conducted in the absence of any commercial or financial relationships that could be construed as a potential conflict of interest.
