# Supplementary material for: Dual-Task Interference in a Simulated Driving Environment: Serial or Parallel Processing?
Source: Front Psychol. 2021 Jan 12;11:579876. doi: 10.3389/fpsyg.2020.579876 (PMC7873965; doi:10.3389/fpsyg.2020.579876)
Supplement: Supplementary file 1 [file Table_1.docx]

**Supplementary Tables and Figures**

**Table S1.** Results of three-way repeated-measure ANOVAs for the effect of task condition (dual vs. single), task order (first vs. second), SOA, and their interaction on RTs of the lane change and image discrimination tasks and Greenhouse-Geisser correction was performed when necessary (indicated by a star).

|  |  | *Cond* | *Order* | *SOA* | *Cond & Order* | *Cond & SOA* | *Order & SOA* | *Cond & Order & SOA* |
| --- | --- | --- | --- | --- | --- | --- | --- | --- |
| *Lance change* | *p* | ***< 0.001*** | *0.59* | ***< 0.001*** | ***0.002*** | ***< 0.001*** | ***< 0.001*** | ***< 0.001*** |
|  | *F* | *28.86* | *0.3* | *42.42* | *13.07* | *46.18* | *50.49* | *19.59* |
|  | *df* | *1,19* | *1,19* | *3,57* | *1,19* | *3,57* | *1.9,36** | *1.7,33** |
|  | $\eta_{p}^{2}$ | *0.6* | *0.01* | *0.69* | *0.40* | *0.70* | *0.72* | *0.50* |
| *Image discrimination* | *p* | ***< 0.003*** | ***0.02*** | ***0.003*** | ***0.008*** | ***< 0.001*** | ***0.001*** | ***0.066*** |
|  | *F* | *18.41* | *6.19* | *5.16* | *8.79* | *9.63* | *8.53* | *2.53* |
|  | *df* | *1,19* | *1,19* | *3,57* | *1,19* | *3,57* | *1.8,34** | *3,57* |
|  | $\eta_{p}^{2}$ | *0.49* | *0.24* | *0.21* | *0.31* | *0.33* | *0.31* | *0.11* |

Cond = task condition (dual vs. single)

**Table S2.** Results of three-way repeated-measure ANOVAs for the effect of task condition (predictable vs. unpredictable), task order (first vs. second), SOA, and their interaction on RTs of the lane change and image discrimination.

|  |  | *Cond* | *Order* | *SOA* | *Cond & Order* | *Cond & SOA* | *Order & SOA* | *Cond & Order & SOA* |
| --- | --- | --- | --- | --- | --- | --- | --- | --- |
| *Lance change* | *p* | *0.46* | *0.91* | ***< 0.001*** | ***< 0.001*** | ***< 0.001*** | ***< 0.001*** | *0.65* |
|  | *F* | *0.54* | *0.01* | *108.4* | *20.26* | *11.65* | *41.95* | *0.54* |
|  | *df* | *1,19* | *1,19* | *3,57* | *1,19* | *3,57* | *1.3,24** | *3,57* |
|  | $\eta_{p}^{2}$ | *0.02* | *0.001* | *0.85* | *0.51* | *0.38* | *0.68* | *0.02* |
| *Image discrimination* | *p* | *0.6* | *0.1* | ***< 0.001*** | ***0.002*** | ***0.003*** | ***0.05*** | *0.38* |
|  | *F* | *0.27* | *2.64* | *10.32* | *13.54* | *6.02* | *3.97* | *0.57* |
|  | *df* | *1,19* | *1,19* | *2,41** | *1,19* | *2,39** | *1.5,30** | *3,57* |
|  | $\eta_{p}^{2}$ | *0.01* | *0.12* | *0.35* | *0.41* | *0.24* | *0.17* | *0.02* |

Cond = task condition (Predictable/Unpredictable)

**Table S3.** Results of three-way repeated-measures ANOVAs for the effect of OP, order and SOA on *v* and *t0* for the lane change and image discrimination.

|  |  | *Cond* | *Order* | *SOA* | *Cond x Order* | *Cond x SOA* | *Order x SOA* | *Cond x Order x SOA* |
| --- | --- | --- | --- | --- | --- | --- | --- | --- |
| *Lance change: drift rate (v)* | *p* | *0.20* | *0.42* | *0.13* | *0.056* | *0.03* | ***< 0.001*** | *0.53* |
|  | *F* | *1.72* | *0.66* | *2.17* | *4.13* | *3.13* | *24.24* | *0.74* |
|  | *df* | *1,19* | *1,19* | *1.7,33** | *1,19* | *3,57* | *3.57* | *3,57* |
|  | $\eta_{p}^{2}$ | *0.08* | *0.03* | *0.1* | *0.17* | *0.14* | *0.56* | *0.03* |
| *Lance change:*  *Non-decision time (t0)* | *p* | *0.34* | *0.10* | ***< 0.001*** | ***< 0.001*** | ***< 0.001*** | ***< 0.001*** | *0.30* |
|  | *F* | *0.96* | *2.87* | *44.96* | *24.6* | *12.13* | *26.84* | *1.22* |
|  | *df* | *1,19* | *1,19* | *2,40** | *1,19* | *1.9,37** | *1.2,23** | *3,57* |
|  | $\eta_{p}^{2}$ | *0.04* | *0.13* | *0.7* | *0.56* | *0.39* | *0.58* | *0.06* |
| *Image discrimination: drift rate (v)* | *p* | *0.60* | *0.13* | *0.67* | *0.38* | ***0.008*** | ***< 0.001*** | *0.40* |
|  | *F* | *0.28* | *249* | *0.5* | *0.79* | *4.64* | *11.48* | *0.98* |
|  | *df* | *1,19* | *1,19* | *3.57* | *1,19* | *3,57* | *3.57* | *3,57* |
|  | $\eta_{p}^{2}$ | *0.01* | *0.11* | *0.02* | *0.04* | *1.96* | *0.37* | *0.04* |
| *Image discrimination: Non-decision time (t0)* | *p* | *0.10* | *0.47* | ***< 0.001*** | *0.02* | *0.10* | *0.57* | *0.33* |
|  | *F* | *2.87* | *0.53* | *13.42* | *5.98* | *2.13* | *0.66* | *1.16* |
|  | *df* | *1,19* | *1,19* | *2,42** | *1,19* | *1.9,36** | *1.8,35** | *3,57* |
|  | $\eta_{p}^{2}$ | *0.13* | *0.02* | *0.4* | *0.23* | *0.1* | *0.03* | *0.05* |

Cond = task condition (Predictable/Unpredictable)

**Table S4.** Results of two-way repeated-measure ANOVAs for the effect of task condition (dual vs. single), SOA, and the interaction between the two on accuracies. All p-values were corrected for multiple comparisons, and Greenhouse-Geisser correction was performed when necessary (indicated by a star).

|  |  | *Lane change-*  *first* | *Lane change-second* | *Image-*  *first* | *Image-second* |
| --- | --- | --- | --- | --- | --- |
| *Task condition (Dual vs. Single)* | *F* | *0.005* | *2.22* | *0.45* | *3.69* |
|  | *df* | *1,19* | *1,19* | *1,19* | *1,19* |
|  | *p* | *0.94* | *0.3* | *0.66* | *0.28* |
|  | $\eta_{p}^{2}$ | *0.0001* | *0.1* | *0.02* | *0.16* |
| *SOA* | *F* | *8.37* | *1.0* | *16.55* | *1.03* |
|  | *df* | *1.6, 30.5** | *3,57* | *3,57* | *3,57* |
|  | *p* | ***0.008*** | *0.39* | ***0.004*** | *0.39* |
|  | $\eta_{p}^{2}$ | *0.3* | *0.05* | *0.46* | *0.05* |
| *Task condition × SOA* | *F* | *0.31* | *1.06* | *2.78* | *1.12* |
|  | *df* | *3,57* | *1.9, 44** | *2.4, 37** | *3,57* |
|  | *p* | *0.81* | *0.49* | *0.24* | *0.49* |
|  | $\eta_{p}^{2}$ | *0.01* | *0.05* | *0.1* | *0.05* |

**Table S5.** Results of two-way repeated-measure ANOVAs for the effect of task condition (predictable vs. unpredictable), SOA, and the interaction between the two on accuracies. All p-values were corrected for multiple comparisons, and Greenhouse-Geisser correction was performed when necessary (indicated by a star).

|  |  | *Lane change-*  *first* | *Lane change-second* | *Image-*  *first* | *Image-second* |
| --- | --- | --- | --- | --- | --- |
| *Task condition (Predictable vs. Unpredictable)* | *F* | *28.13* | *5.1* | *1.94* | *3.6* |
|  | *df* | *1, 19* | *1, 19* | *1, 19* | *1, 19* |
|  | *p* | ***< 0.001*** | *0.072* | *0.17* | *0.09* |
|  | $\eta_{p}^{2}$ | *0.59* | *0.21* | *0.09* | *0.15* |
| *SOA* | *F* | *2.85* | *1.51* | *3.33* | *0.45* |
|  | *df* | *2, 38** | *2, 41** | *2, 38** | *3, 57* |
|  | *p* | *0.12* | *0.29* | *0.12* | *0.69* |
|  | $\eta_{p}^{2}$ | *0.13* | *0.07* | *0.14* | *0.02* |
| *Task condition × SOA* | *F* | *2.85* | *1.51* | *4.1* | *0.1* |
|  | *df* | *2, 38** | *2, 41** | *2, 54** | *3, 57* |
|  | *p* | *0.12* | *0.29* | ***0.04*** | *0.95* |
|  | $\eta_{p}^{2}$ | *0.13* | *0.07* | *0.17* | *0.005* |

**Table S6.** Results of the comparison of the dual-task effect across the two tasks. For this analysis, first, we subtracted RTs of the single-task condition from the RTs of the dual-task condition for each task (when it was presented second) and for each SOA. We then ran a two-way repeated measure ANOVA with task (driving/image) and SOA as factors. As can be seen in the following table, the main effect of the task was not significant *(p = 0.69)*, but the effect of SOA (*p < 0.001)* and its interaction with the task *(p = 0.001)* was significant. To further examine the effect of the interaction of task and SOA, we used a paired sample t-test. Results of the pairwise comparisons indicated that the difference in the magnitude of interference was only significant for SOA 600 (t(1,19) = -2.89, *p* = 0.009). As can be seen in Fig. 3, the driving RT in the SOA 600 was the same for the dual- and single-task conditions, while for the image task, the dual-task RT was longer than the single-task. This might be related to the higher priority of the driving task causing the resources to not be fully devoted to the image task when it was presented first even at the largest SOA.

|  | *Task condition* | *SOA* | *Task condition x SOA* |
| --- | --- | --- | --- |
| *F* | *0.164* | *30.03* | *6.49* |
| *df* | *1, 19* | *3, 57* | *3, 57* |
| *p* | *0.69* | ***< 0.0001*** | ***0.001*** |
| $\boldsymbol{\eta}_{\boldsymbol{p}}^{\boldsymbol{2}}$ | *0.009* | *0.61* | *0.25* |

**Table S7.** Results of pairwise comparisons of RTs across the two task conditions (single vs. dual / predictable vs. unpredictable) for each SOA. All p-values are corrected for multiple comparisons across time bins using FDR at q < 0.05.

| *Task Condition* | *SOA* |  | *Lane change-*  *first* | *Lane change-second* | *Image-first* | *Image-second* |
| --- | --- | --- | --- | --- | --- | --- |
| *Dual vs. Single* | *30* | *t (1,19)* | *2.14* | *8.01* | *1.14* | *5.01* |
|  |  | *p* | ***0.04*** | ***< 0.001*** | *0.26* | ***< 0.001*** |
|  | *100* | *t (1,19)* | *2.64* | *7.04* | *1.91* | *5.82* |
|  |  | *p* | ***0.03*** | ***< 0.001*** | *0.09* | ***< 0.001*** |
|  | *300* | *t (1,19)* | *2.16* | *5.77* | *3.98* | *2.20* |
|  |  | *p* | ***0.04*** | ***< 0.001*** | ***0.003*** | ***0.03*** |
|  | *600* | *t (1,19)* | *4.77* | *-1.03* | *1.96* | *2.67* |
|  |  | *p* | ***< 0.001*** | *0.31* | *0.09* | ***0.02*** |
| *Predictable vs. Unpredictable* | *30* | *t (1,19)* | *-3.20* | *4.30* | *-2.56* | *2.0* |
|  |  | *p* | ***0.01*** | ***0.001*** | ***0.04*** | *0.12* |
|  | *100* | *t (1,19)* | *-3.99* | *2.94* | *-2.55* | *2.93* |
|  |  | *p* | ***0.004*** | ***0.01*** | ***0.04*** | ***0.03*** |
|  | *300* | *t (1,19)* | *-2.55* | *1.84* | *-0.87* | *1.54* |
|  |  | *p* | ***0.02*** | *0.1* | *0.52* | *0.18* |
|  | *600* | *t (1,19)* | *-1.60* | *0.11* | *-0.63* | *0.78* |
|  |  | *p* | *0.12* | *0.9* | *0.53* | *0.46* |

**Table S8.** Results of pairwise comparisons of accuracies across the two task conditions (single vs. dual/predictable vs. unpredictable) for each SOA. All p-values are corrected for multiple comparisons across time bins using FDR at q < 0.05.

| *Task Condition* | *SOA* |  | *Lane change-*  *first* | *Lane change-second* | *Image-first* | *Image-second* |
| --- | --- | --- | --- | --- | --- | --- |
| *Dual vs. Single* | *30* | *t (1,19)* | *-3.21* | *-1.00* | *-3.18* | *-1.41* |
|  |  | *p* | ***0.02*** | *0.33* | ***0.02*** | *0.17* |
|  | *100* | *t (1,19)* | *-1.34* | *-1.74* | *-0.55* | *-0.98* |
|  |  | *p* | *0.19* | *0.19* | *0.77* | *0.33* |
|  | *300* | *t (1,19)* | *-2.03* | *-1.96* | *-0.13* | *-0.78* |
|  |  | *p* | *0.07* | *0.19* | *0.89* | *0.44* |
|  | *600* | *t (1,19)* | *-2.05* | *-1.00* | *-0.76* | *-0.96* |
|  |  | *p* | *0.07* | *0.33* | *0.77* | *0.34* |
| *Predictable vs. Unpredictable* | *30* | *t (1,19)* | *0.12* | *-* | *-1.41* | *1.42* |
|  |  | *p* | *0.99* | *-* | *0.3* | *0.22* |
|  | *100* | *t (1,19)* | *1.14* | *-0.85* | *1.91* | *0.43* |
|  |  | *p* | *0.26* | *0.54* | *0.28* | *0.67* |
|  | *300* | *t (1,19)* | *-0.48* | *-1.4* | *0.23* | *2.08* |
|  |  | *p* | *0.63* | *0.52* | *0.81* | *0.17* |
|  | *600* | *t (1,19)* | *0.56* | *0.63* | *1.23* | *1.8* |
|  |  | *p* | *0.58* | *0.54* | *0.3* | *0.17* |

**Table S9.** Results of pairwise comparisons of drift rates (v) and non-decision times (t0) across the two task conditions (predictable vs. unpredictable) for each SOA. All p-values are corrected for multiple comparisons across time bins using FDR at q < 0.05.

| *Task Condition* | *SOA* |  | *Lane change-*  *first* | *Lane change-second* | *Image-first* | *Image-second* |
| --- | --- | --- | --- | --- | --- | --- |
| *Drift rate (v)*  *(Predictable vs. Unpredictable)* | *30* | *t (1,19)* | *0.83* | *-1.85* | *0.56* | *-0.7* |
|  |  | *p* | *0.41* | *0.15* | *0.65* | *0.48* |
|  | *100* | *t (1,19)* | *3.54* | *2.13* | *2.35* | *-1.41* |
|  |  | *p* | ***0.008*** | *0.15* | *0.11* | *0.48* |
|  | *300* | *t (1,19)* | *0.93* | *0.36* | *0.44* | *1.18* |
|  |  | *p* | *0.41* | *0.74* | *0.65* | *0.48* |
|  | *600* | *t (1,19)* | *0.88* | *-1.04* | *-0.71* | *0.86* |
|  |  | *p* | *0.41* | *0.41* | *0.65* | *.48* |
| *Non-decision time (t0)*  *(Predictable vs. Unpredictable)* | *30* | *t (1,19)* | *-2.51* | *3.66* | *-1.02* | *2.97* |
|  |  | *p* | *0.08* | ***0.004*** | *0.47* | ***0.04*** |
|  | *100* | *t (1,19)* | *-1.57* | *3.66* | *1.07* | *1.73* |
|  |  | *p* | *0.26* | ***0.004*** | *0.47* | *0.19* |
|  | *300* | *t (1,19)* | *- 0.12* | *2.52* | *0.94* | *1.15* |
|  |  | *p* | *0.89* | ***0.028*** | *0.47* | *0.34* |
|  | *600* | *t (1,19)* | *-0.68* | *-0.32* | *-0.31* | *0.6* |
|  |  | *p* | *0.67* | *0.75* | *0.75* | *0.55* |


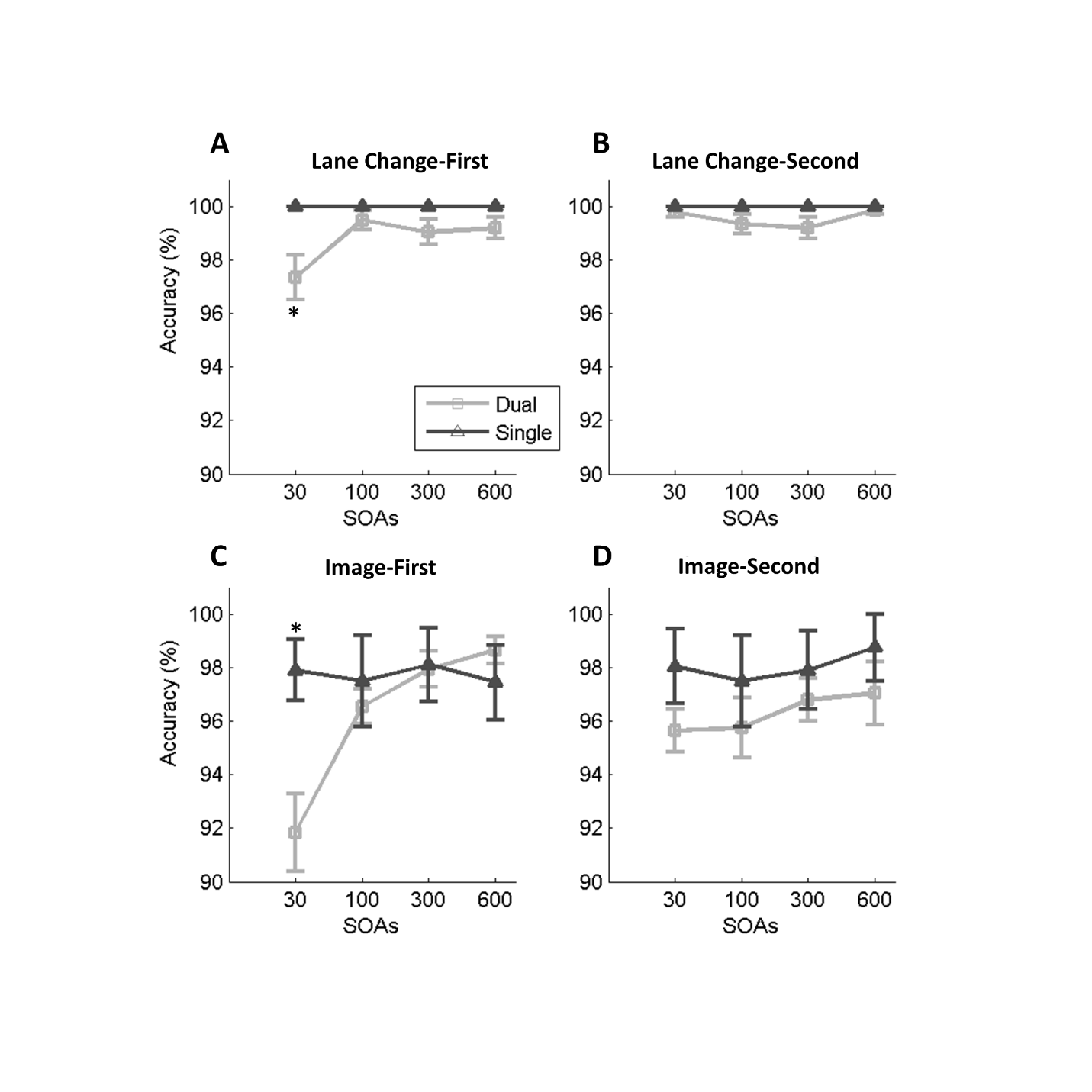


**Fig. S1.** Effect of task condition (dual vs. single) and SOA on accuracies. **(A, B)** These panels indicate the accuracies for the lane change turn in the lane change-first and lane change-second task orders, respectively, for the single-task (dark grey) and the dual-task (light grey) conditions. **(C, D)** These panels show the image discrimination accuracies in the single (dark grey) and the dual (light grey) task conditions for the image-first and the image-second task orders, respectively. In all panels, error bars show standard errors of mean and star shows a significant difference between task conditions for each SOA (* < 0.05).


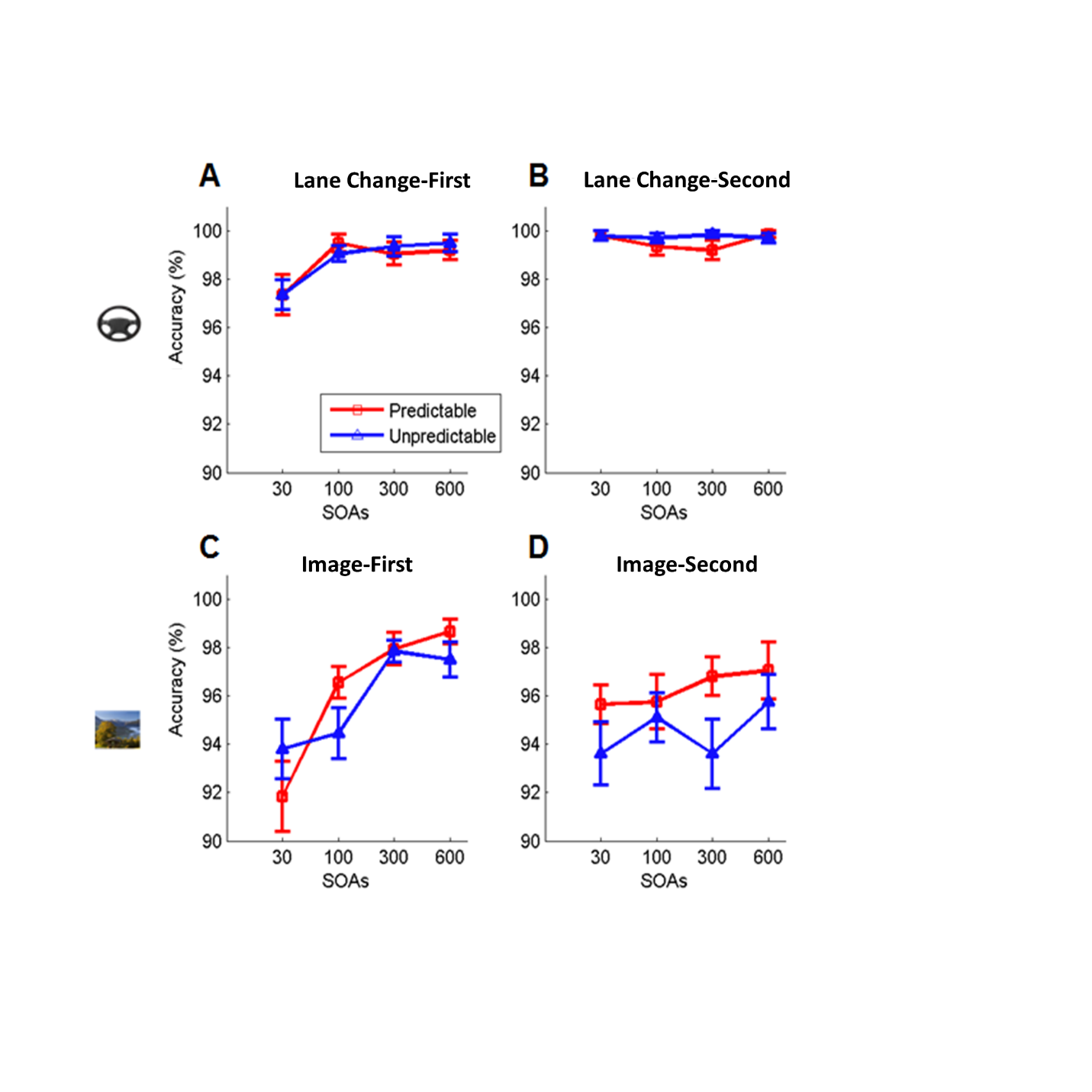


**Fig. S2.** Effect of OP and SOA on accuracies. The two top panels show the accuracies for the driving turn in the lane change-first (A) and lane change-second (B) task orders for the predictable (red) and the unpredictable (blue) task order conditions. The two bottom panels show the accuracies for the image discrimination task in the image-first (C) and image-second (D) task orders for the predictable (red) and the unpredictable (blue) task order conditions. In all panels, error bars show standard errors of mean.


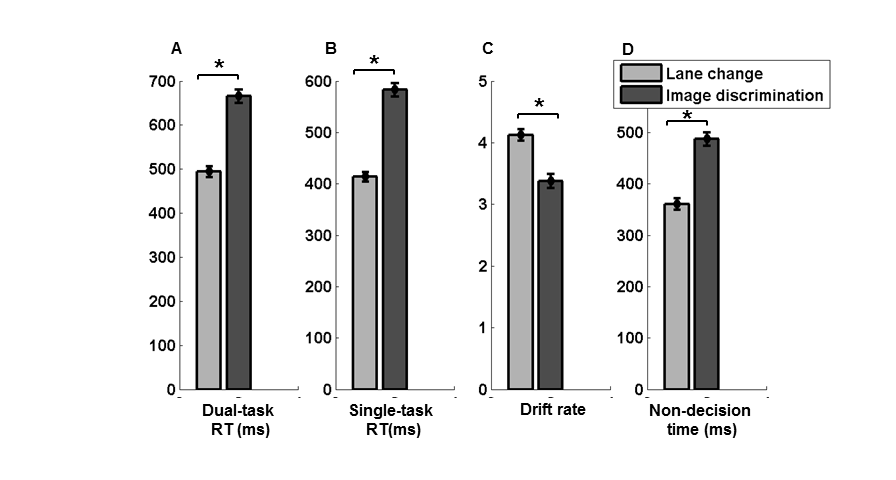


**Fig S3.** Comparison of RTs, drift rate, and non-decision time between the two tasks. **(A, B)** The mean RTs for the dual-task and single-task conditions, respectively, for the lane change task (light grey) and the image discrimination task (dark grey). **(C)** The mean drift rate for the lane change task (light grey) and the image discrimination task (dark grey). **(D)** The mean non-decision time for the lane change task (light grey) and the image discrimination task (dark grey). First, we averaged the RTs, drift rates, and non-decision times across SOA for each task, separately and then, we compared the results. We observed the RT and t0 were significantly longer for the image discrimination than the lane change task (*RT: t(1,19) = -11.58, p < 0.001; t0: t(1,19) = -10.44, p < 0.001* ), but the v was significantly higher for lane change task than the image discrimination task (*t(1,19) = 7.23, p < 0.001*). These results indicate, in general, the RT, decision time (related to the inverse of v) and non-decision time of image discrimination task were longer than the lane change task.
